# Dietary Virgin Olive Oil Reduces Blood Brain Barrier Permeability, Brain Edema, and Brain Injury in Rats Subjected to Ischemia-Reperfusion

**DOI:** 10.1100/tsw.2010.128

**Published:** 2010-06-29

**Authors:** Fatemeh Mohagheghi, Mohammad Reza Bigdeli, Bahram Rasoulian, Ali Asghar Zeinanloo, Ali Khoshbaten

**Affiliations:** ^1^ Faculty of Biological Sciences, Shahid Beheshti University, G.C. Tehran, Iran; ^2^ Razi Herbal Medicines Research Center, Lorestan University of Medical Sciences, Khorramabad, Iran; ^3^ Seed and Plant Improvement Institute, Karaj, Iran; ^4^ Research Center for Chemical Injuries, Baqiyatallah Medical Sciences University, Tehran, Iran

**Keywords:** neuroprotective, virgin olive oil, focal cerebral ischemia, infarct volume, edema, blood brain barrier permeability

## Abstract

Recent studies suggest that dietary virgin olive oil (VOO) reduces hypoxia-reoxygenation injury in rat brain slices. We sought to extend these observations in an *in vivo* study of rat cerebral ischemia-reperfusion injury. Four groups, each consisting of 18 Wistar rats, were studied. One group (control) received saline, while three treatment groups received oral VOO (0.25, 0.5, and 0.75 mL/kg/day, respectively). After 30 days, blood lipid profiles were determined, before a 60-min period of middle cerebral artery occlusion (MCAO). After 24-h reperfusion, neurological deficit scores, infarct volume, brain edema, and blood brain barrier permeability were each assessed in subgroups of six animals drawn from each main group. VOO reduced the LDL/HDL ratio in doses of 0.25, 0.5, and 0.75 mL/kg/day in comparison to the control group (*p* < 0.05), and offered cerebroprotection from ischemia-reperfusion. For controls vs. doses of 0.25 vs. 0.5 vs. 0.75 mL/kg/day, attenuated corrected infarct volumes were 207.82 ± 34.29 vs. 206.41 ± 26.23 vs. 124.21 ± 14.73 vs. 108.46 ± 31.63 mm^3^; brain water content of the infarcted hemisphere was 82 ±± 0.25 vs. 81.5 ± 0.56 vs. 80.5 ± 0.22 vs. 80.5 ± 0.34%; and blood brain barrier permeability of the infarcted hemisphere was 11.31 ± 2.67 vs. 9.21 ± 2.28 vs. 5.83 ± 1.6 vs. 4.43 ± 0.93 µg/g tissue (*p* < 0.05 for measures in doses 0.5 and 0.75 mL/kg/day vs. controls). Oral administration of VOO reduces infarct volume, brain edema, blood brain barrier permeability, and improves neurologic deficit scores after transient MCAO in rats.

## INTRODUCTION

Brain ischemia induces the release of excitatory amino acids, with subsequent receptor activation leading to calcium influx, metabolic and electrophysiological dysfunction, and oxidative stress (including lipid peroxidation)[1]. Subsequent reperfusion worsens this oxidative stress, potentiating ischemic injury[2]. Diets rich in antioxidants might thus offer neuroprotection in cases of ischemic stroke.

The "Mediterranean diet" is powerfully antioxidant, and a number of international scientific organizations now recommend a modified version[3] to prevent conditions in which oxidative stress may play an etiological role[4]. A key component of this diet is olive oil, which contains monounsaturated fatty acids (MUFA) and polyphenols, compounds with a clear antioxidant effect[5]. Tyrosol and hydroxytyrosol (HT) are two such olive oil phenolic compounds; HT administration enhances plasma antioxidant capacity and lowers production of proinflammatory and prothrombotic mediators in laboratory animals[6].

An HT extract prepared from olive mill wastewater reduces neuronal damage induced by chemical oxidative stress (from ferrous salts or sodium nitroprusside administration)[7]. Meanwhile, tyrosol has neuroprotective effects in a model of transient focal cerebral ischemia in rats[8], and both HT and HT acetate protect brain slices subjected to hypoxia-reoxygenation[9].

Thus, while past studies suggest that virgin olive oil (VOO) may protect brain slices from oxidative stress[9,10], *in vivo* neuroprotective effects from ischemia-reperfusion injury have yet to be clarified. We thus sought to characterize the impact of dietary VOO on brain infarct volume, brain edema, blood brain barrier permeability, and neurological dysfunction resulting from transient middle cerebral artery occlusion (MCAO) in rats.

## METHOD

All experimental animal procedures were conducted with the approval of the Ethics Committee of Shahid Beheshti University of Iran.

### Materials

Olive oil was industrially extracted. Olives (*Olea europaea* L., Zard variety) were crushed using a hammer crusher, malaxation was performed at 25°C, and the oil was extracted by centrifugation (10,000 × *g*; 10 min) using a decanter at a low level of water addition.

Acidity was measured in an approved fashion (ISO/FDIS 660). Peroxide content was determined as described in ISO 3960:2001 and expressed as milliequivalents (mequiv) of active oxygen per kilogram of oil. Fatty acids were analyzed by gas chromatography (GC: Varian 3800, Australia) to identify and quantify the individual fatty acid in the oils, using a method adapted from ISO 5508:1990 (Table 1). Phenolics were separated by reversed-phase high-performance liquid chromatography (HPLC: Yonglin, South Korea) as described in the CISC definition. Tocopherols (α-tocopherol) were analyzed by HPLC as described in ISO 9936:1997. Sterols were analyzed by GC (Yonglin, South Korea) according to the ISO 5725-1:1994. The composition of the VOO administered to rats is shown in Table 1.

### Animals

Male Wistar rats (body weight 200–300 g) were housed under conditions of controlled temperature (22 ± 2°C) and constant humidity, with 12 h light/dark cycle (light on 07:00–19:00). Food and water were available *ad libitum*.

Four groups, each of 18 animals, received a dietary intervention (10–11.00 h daily) for 30 days. A control received gastric gavage (g.g.) with daily saline. The other three groups received 0.25, 0.5, and 0.75 mL/kg/day g.g. of the same VOO doses identified using previously published data[10] and by interpolation to healthy humans (25 mL/day). Two hours after the last dose, the animals were subjected to 60 min of MCAO. Twenty-four hours later, neurobehavioral studies were performed before each group

**TABLE 1**
**Main Composition of the VOO Administered to Rat Groups**

| | |
|---|---|
| Fatty acids (%) | |
| C16:0 | 15.99 |
| C16:1 | 1.024 |
| C18:0 | 1.815 |
| C18:1 | 68.822 |
| C18:2 | 9.951 |
| C18:3 | 0.602 |
| C20:0 | 0.383 |
| C20:1 | 0.320 |
| C22:0 | 0.105 |
| C24:0 | 0.979 |
| Total | 100 |
| Acidity (%) | 0.28 |
| Peroxide value (meq$O^2$/kg oil) | 0.34 |
| Extinction coefficient in K232 | 2.54 |
| Extinction coefficient in K270 | 0.07 |
| Minor component (mg/kg) | |
| α-Tocopherol | 210 |
| Total polyphenols | 320 |
| Cholesterol | 0.00054 |
| β-Sitosterol | 1.332 |

was split into three subgroups of six, and sacrificed for study of infarct volume, brain edema, and blood brain barrier (BBB) permeability, respectively. In addition, another group (12 animals) was managed according to the control protocol, but underwent surgery without MCAO. When sacrificed, these sham-operated animal groups were divided into subgroups (n = 6 in each) for evaluation of brain edema and BBB permeability, respectively.

## Assessment of Physiological Variables

Oxygen saturation ($So_2$; pulse oximeter, Radiometer, Denmark), heart rate and blood pressure (PowerLab, ADInstruments, Australia), weight, and respiration rate were measured pre- (first day) and post- ($30^{th}$ day) treatment in 0, 0.25, 0.5, and 0.75 mL/kg/day VOO groups.

## Lipid Profiles

At the end of day 30, before surgery, all animals were anesthetized with chloral hydrate (400 mg/kg i.p.). A venous blood sample was drawn and centrifuged at 7,000 × *g* for 10 min, and the supernatant (serum) frozen at –20°C until assayed for HDL, triglyceride, and cholesterol content (Pars Azmun, Iran according to manufacturer's instructions) and autoanalyzed (Liasys, Roma, Italy).

## MCAO

The rats were weighed and intraperitoneally (i.p.) anesthetized with chloral hydrate (Merck, Germany) (400 mg/kg). MCAO was performed as described by Longa et al.[11]. Briefly, using microscopic surgery, a 3-0 silicone-coated nylon suture was introduced through the external carotid artery stump. The occluder was advanced into the internal carotid artery 20–22 mm beyond the carotid bifurcation until mild resistance indicated that the tip was lodged in the anterior cerebral artery and blocked the blood flow to the middle cerebral artery. Reperfusion was started by withdrawing the suture after 60 min of ischemia. Rectal temperature was monitored (Citizen-513w) and maintained at 37.0°C by surface heating and cooling during surgery. A surface laser Doppler flowmeter (MBF3D, Moor Instruments, U.K.) was used to record regional cerebral blood flow (rCBF). Using a stereotaxic device and a low-speed dental drill, a burr hole of 2 mm in diameter was made over the skull at 1 mm posterior and 5 mm lateral to the bregma on the right side. A needle-shaped laser probe was placed on the dura away from visible cerebral vessels. Steady-state baseline values were recorded before MCAO so that all rCBF data were expressed as percentages of the respective basal value[12]. Doppler flux was continuously measured from 30 min before MCAO to 30 min after reperfusion.

## Assessment of Neurobehavioral Impact

After the suture was withdrawn, the rats were returned to their separate cages. Twenty-four hours later, the rats were assessed neurologically by an observer who was blind to the animal groups. The neurobehavioral scoring was performed using a six-point scale as previously described[11]: normal motor function = 0; flexion of contralateral forelimb upon suspension vertically by tail or failure to extend forepaw = 1; circling to the contralateral side, but have normal posture at rest = 2; loss of righting reflex = 3; no spontaneous motor activity = 4. Death was considered as score 5 only when a large infarct volume was present in the absence of subarachnoid hemorrhage. If the rats died due to subarachnoid hemorrhage or pulmonary insufficiency and asphyxia, they were eliminated from the study.

## Assessment of Infarct Volume

After sacrifice with chloral hydrate (800 mg/kg), the animals were decapitated and the brains rapidly removed and cooled in saline at 4°C for 15 min. Eight 2-mm-thick coronal sections were then cut (Brain Matrix, Iran), beginning at the olfactory bulb. The slices were immersed in 2% 2,3,5-triphenyltetrazolium chloride solution (Merck, Germany) and kept at 37°C in a water bath for 15 min. The slices were then digitally photographed (Lumix-Panasonic camera, Japan connected to a computer). Unstained areas were defined as infarct and were measured using image analysis software (UTHSCSA Image Tool). The infarct volume was calculated by measuring the unstained and stained areas in each hemisphere slice, multiplying this by slice thickness (2 mm), and then summing all of the eight slices according to the method of Swanson et al.[13]: corrected infarct volume = left hemisphere volume − (right hemisphere volume − infarct volume).

## Assessment of Brain Water Content

After decapitation, the brain was removed. The cerebellum, pons, and olfactory bulb were separated and their wet weights (WW) measured[14]. Dry weights (DW) were assessed after 24 h at 120°C. Brain water content (BWC) was calculated as $[(WW - DW)/WW] \times 100$.

## Assessment of Blood Brain Barrier Integrity

The integrity of BBB was evaluated by studying Evans Blue (EB, Sigma Chemicals, U.S.) extravasation. Briefly, the rats received 4 mL/kg of 2% EB solution in saline by tail vein injection 30 min after MCAO. Twenty-four hours after reperfusion, the thoracic cavity was opened under anesthesia. The rats were perfused with 250 mL saline transcardially to wash out intravascular EB until colorless perfusion fluid was obtained from the atrium. After decapitation, the brains were removed and the hemispheres separated and weighed. The right and left hemispheres were separately homogenized in 2.5-mL phosphate-buffered saline to extract the EB and to precipitate protein, 2.5 mL of 60% trichloroacetic acid was added and mixed by vortex for 3 min. The samples were then placed at 4°C for 30 min and centrifuged for 30 min at 1,000 × *g*. The amount of EB in the supernatants was measured at 610 nm using a spectrophotometer (Perkin-Elmer, U.S.). EB levels were expressed as µg/g of brain tissue against a standard curve[14].

## Statistical Analysis

Physiological variables were assessed by paired-samples T test. Infarct volumes, brain water content, EB extravasation, blood flow, body weight, cholesterol, triglyceride, LDL, HDL, and VLDL were compared using one-way ANOVA (SPSS 11.0; post hoc LSD). The neurologic deficit scores (NDS) were analyzed using the Mann-Whitney U test. Data were expressed as means ± SEM. A value of $p < 0.05$ was considered significant.

## RESULTS

Physiological variable changes in the VOO animals were not different from that in control animals (Table 2). In each group, MCAO reduced cerebral blood flow to less than 24% of that at baseline (Fig. 1).

**TABLE 2**
**Physiological Variables in Animals Treated with VOO (0, 0.25, 0.5, and 0.75 mL/kg for 30 days)**

| Physiological Variables | Pretreatment | | | | Post-Treatment | | | |
|---|---|---|---|---|---|---|---|---|
| | 0 | 0.25 | 0.5 | 0.75 | 0 | 0.25 | 0.5 | 0.75 |
| $S_{O2}$ (%) | 85 ± 1.17 | 83.66 ± 1.35 | 84.66 ± 1.48 | 85 ± 1.01 | 83.33 ± 0.89 | 84 ± 1.76 | 84.33 ± 1.15 | 82 ± 0.58 |
| Heart rate | 372.66 ± 25.35 | 392.66 ± 37.59 | 370.66 ± 30.39 | 406.33 ± 42.02 | 352 ± 33.75 | 413.66 ± 11.01 | 397.66 ± 29.53 | 385 ± 39.66 |
| Blood pressure (mmHg) | 89.66 ± 7.81 | 91.33 ± 4.41 | 86 ± 11.48 | 90.66 ± 4.7+9 | 82.33 ± 10.29 | 87.5 ± 2.94 | 90.33 ± 12.48 | 95 ± 2.54 |
| Weight (g) | 229.72 ± 3.02 | 259.94 ± 4.1 | 257.72 ± 4.15 | 232.5 ± 2.86 | 261.38 ± 3.52 | 285.88 ± 5.4 | 291.16 ± 4.44 | 264.88 ± 3.58 |
| Respiration rate (Hz) | 1.42 ± 0.08 | 1.41 ± 0.04 | 1.53 ± 0.15 | 1.49 ± 0.05 | 1.48 ± 0.08 | 1.38 ± 0.05 | 1.42 ± 0.08 | 1.6 ± 0.16 |

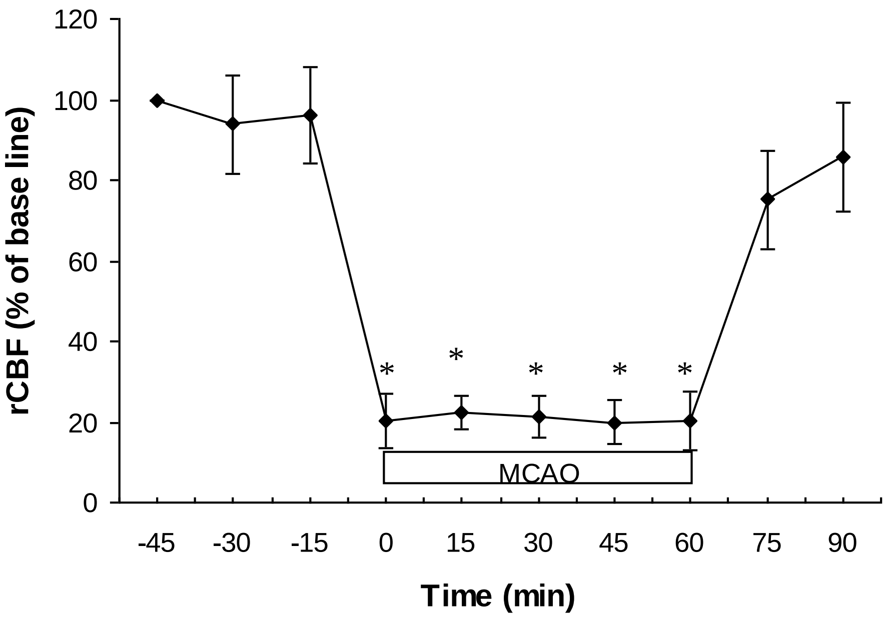

**FIGURE 1**. rCBF before and during MCAO, and after reperfusion (*; $p$ = 0.000, n = 6).

While serum cholesterol levels in the 0.5 mL/kg/day group were similar to that in controls, they were higher in the 0.25 and 0.75 mL/kg/day group ($p$ = 0.032 and $p$ = 0.002, respectively, Fig. 2). Serum triglyceride levels were greater in the 0.25, 0.5, and 0.75 mL/kg/day VOO groups when compared to controls ($p$ = 0.008, $p$ = 0.011, and $p$ = 0.000, respectively). The HDL levels in the control group were 6 ± 0.36 mg/dL, but higher in the 0.25, 0.5, and 0.75 mL/kg/day VOO groups (11.33 ± 1.47, 13.33 ± 2.51, and 19 ± 1.54 mg/dL; $p$ = 0.034, $p$ = 0.005, and $p$ = 0.000, respectively, Fig. 2). VOO significantly increased the VLDL levels in the 0.25, 0.5, and 0.75 mL/kg/day groups ($p$ = 0.009, $p$ = 0.012, and $p$ = 0.000, respectively, Fig. 2). The LDL levels were similar in the control and all VOO groups. The LDL/HDL ratio was 9.41 ± 0.9 in the control group, but 4.95 ± 1.04, 4.21 ± 0.91, and 2.25 ± 0.76 in the 0.25, 0.5, and 0.75 mL/kg/day groups, respectively ($p$ = 0.003, $p$ = 0.001, and $p$ = 0.000, Fig. 2).

Median NDS were reduced by VOO, being 2 (range: 0–4), 1 (range: 0–3), 1 (range: 0–3), and 2 (range: 1–4) in the 0.25, 0.5, 0.75 mL/kg/day, and control group, respectively (Table 3). The putative beneficial effects of VOO were confirmed by a reduction in infarct volume (Fig. 3).

Pretreatment with 0.5 and 0.75 mL/kg/day VOO for 30 days and 2 h before MCAO resulted in a reduction of infarct volume, while the lower dose (0.25 mL/kg/day) had no effect (Fig. 3). The neuroprotection exerted by VOO was mainly seen in the penumbra (cortex), while it was slightly observed in the infarct core.

Focal cerebral ischemia significantly increased the brain water content in the ischemic hemisphere in control groups ($p$ = 0.000). The dose of 0.5 and 0.75 mL/kg/day reduced the postischemic brain water content increment ($p$ = 0.006), while the lower dose (0.25 mL/kg/day) was without effect (Fig. 4).

Brain edema formation was associated with increased BBB permeability at 24 h. In the control group, EB concentration in ischemic cerebral tissue was 11.31 ± 2.67 and 3.83 ± 0.95 µg/g tissue in the nonischemic left hemisphere. Administration of VOO at doses of 0.5 and 0.75 mL/kg/day was associated with a reduction in EB extravasation in the ischemic brain (the dose of 0.5 mL/kg/day: left hemisphere = 2.68 ± 0.33, right hemisphere = 5.83 ± 1.6 µg/g tissue; the dose of 0.75 mL/kg/day: left hemisphere = 2.31 ± 0.31, right hemisphere = 4.43 ± 0.93 µg/g tissue). BBB permeability in the contralateral hemisphere was not significantly affected in the brain pretreated with VOO (Fig. 5). This effect was not observed at the lower VOO dose of 0.25 mL/kg/day: left hemisphere = 3 ± 0.47, right hemisphere = 9.21 ± 2.28 µg/g tissue.

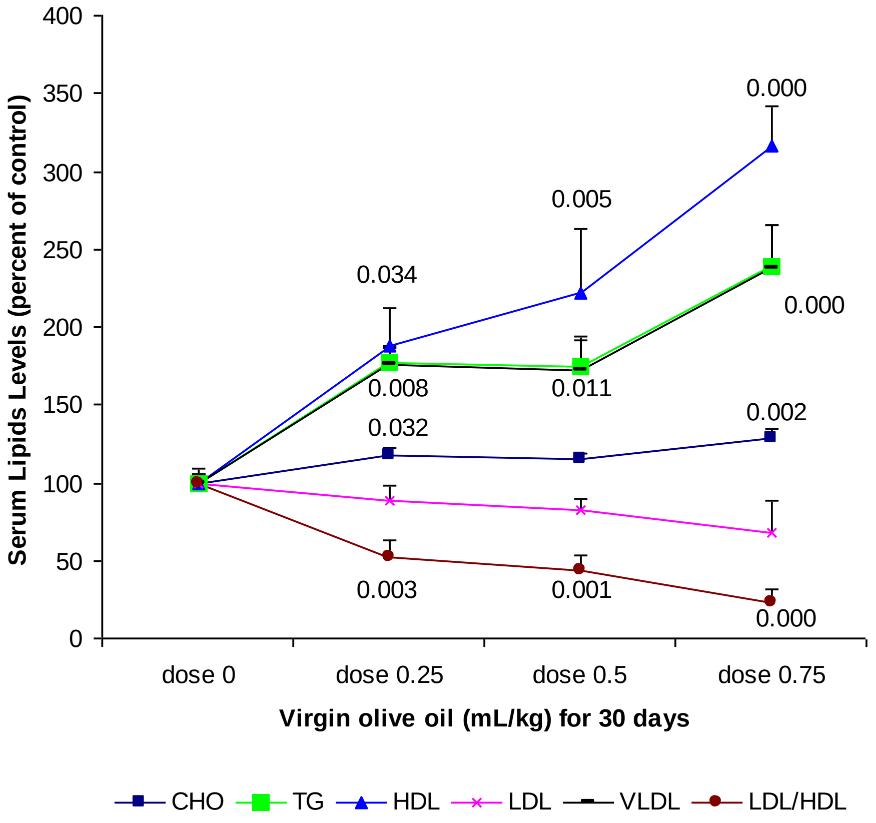

**FIGURE 2.** Serum lipid levels at the end of VOO pretreatment. VOO increased cholesterol levels by pretreatment with dose 0.25 and 0.75 mL/kg/day, and triglyceride, HDL, and VLDL levels at 0.25, 0.5, and 0.75 mL/kg/day. VOO decreased LDL/HDL levels at 0.25, 0.5, and 0.75 mL/kg/day ($p < 0.05$; n = 6).

**TABLE 3**
**Distribution of NDS in Each Group**

| Code | Experimental Groups | NDS in Each Group | | | | | Total (N) | Median | Statistical Results (*p* Value) |
|---|---|---|---|---|---|---|---|---|---|
| | | 0 | 1 | 2 | 3 | 4 | | | |
| 1 | Dose 0 | 0 | 3 | 11 | 2 | 2 | 18 | 2 | 1:3 sig. (0.003) |
| 2 | Dose 0.25 | 1 | 4 | 11 | 1 | 1 | 18 | 2 | 1:4 sig. (0.002) |
| 3 | Dose 0.5 | 4 | 8 | 5 | 1 | 0 | 18 | 1 | 2:3 sig. (0.034) |
| 4 | Dose 0.75 | 5 | 7 | 5 | 1 | 0 | 18 | 1 | 2:4 sig. (0.027) |
| — | Total(N) | 10 | 22 | 32 | 5 | 3 | 72 | — | 1:2 and 3:4 nonsig. |

NDS: neurologic deficit score; N: the number of cases in each group; sig: significant; nonsig: nonsignificant.

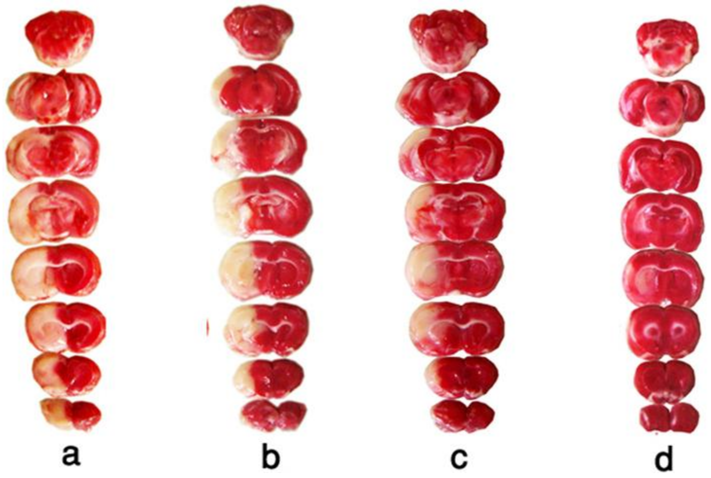

**A**

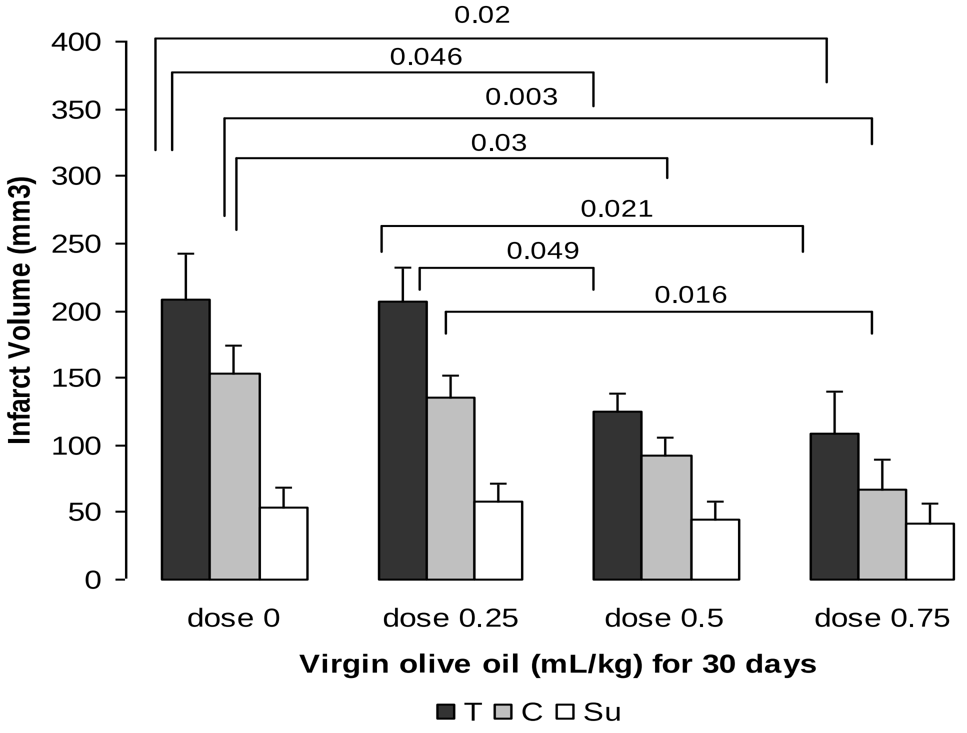

**B**

**FIGURE 3.** (A) Photograph showing the neuroprotective effect of VOO on focal cerebral ischemia. All sections of the brain were stained with TTC at 24 h after 60 min of ischemia. Each column presents the series of rat brain coronal sections: (a) brain sections of control rat; (b,c,d) brain sections of 0.25, 0.5, and 0.75 mL/kg/day pretreated rats, respectively. (B) The graph shows the effects of various doses (0, 0.25, 0.5, and 0.75 mL/kg) of VOO on infarct volume in different experimental groups in cortex (C), subcortex (Su), and total (T) area of brain (each group n = 6).

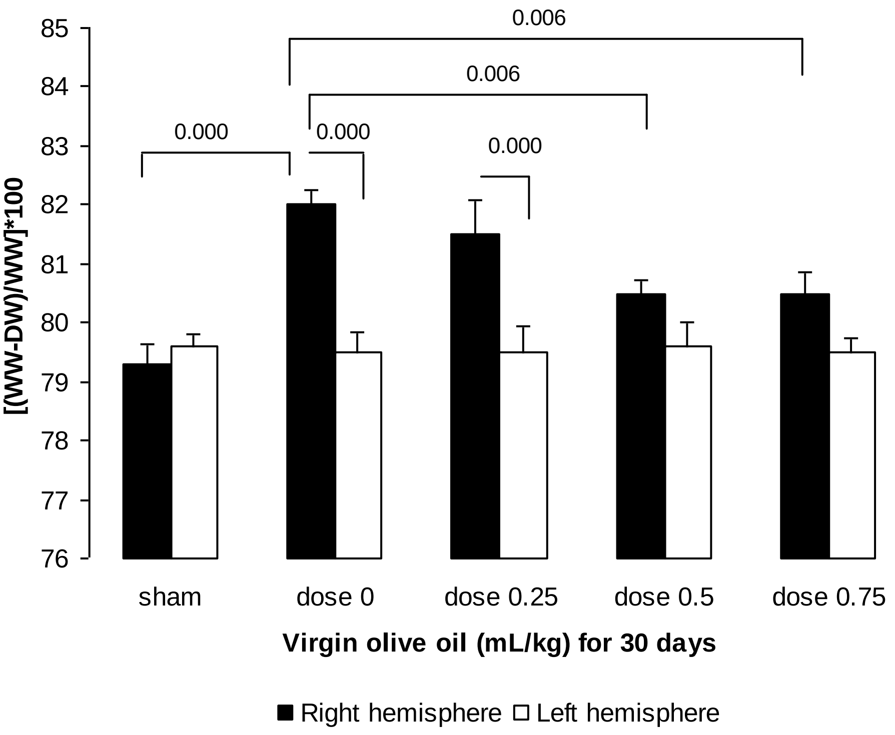

**FIGURE 4.** Brain water content in various experimental groups including left and right hemisphere of sham-operated, 0, 0.25, 0.5, and 0.75 mL/kg of VOO (n = 6).

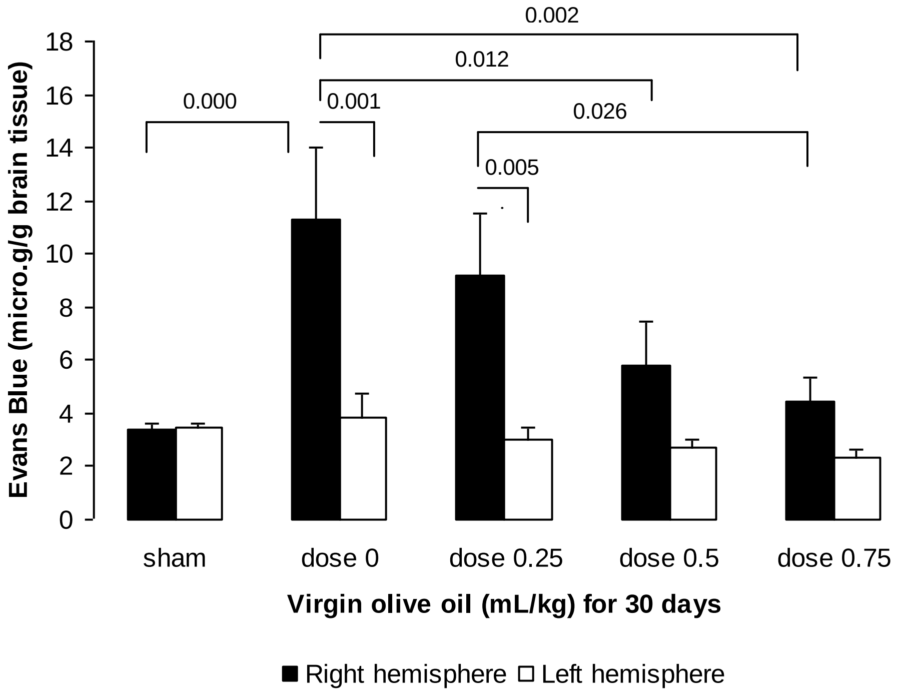

**FIGURE 5.** EB extravasations in various experimental groups including left and right hemisphere of sham-operated, 0, 0.25, 0.5, and 0.75 mL/kg of VOO (n = 6).

## DISCUSSION

Our data suggest that pretreatment with dietary VOO may reduce infarct volume, brain edema, BBB permeability, and neurobehavioral deficit scores in a reliable and reproducible animal model of stroke followed by reperfusion[11]. These data are supported by recent studies suggesting that dietary VOO reduces hypoxia-reoxygenation injury in rat brain slices[9,10]. Our findings indicate that dietary VOO may influence brain water content and brain water homeostasis by increasing BBB integrity. The neuroprotection exerted by VOO was mainly seen in the penumbra (cortex). The lack of protective effects in the subcortical area could be due to the more severe damage in the subcortical area than in the cortex. It has been shown that neuronal injury after transient MCAO is more severe in the subcortex than in the cortex[15].

Although our data suggest that VOO-mediated neuroprotection is due to the reduction of postischemic infarct volume and brain edema, other mechanisms may be at work. Whether the beneficial effects of olive oil on the cardiovascular system are exclusively due to oleic acid is questionable. Indeed, intake of monounsaturated fatty acids (MUFAs) lowers LDL susceptibility to oxidation compared to supplementation with polyunsaturated fatty acids (PUFAs)[16]. Furthermore, oleic acid has been shown to exert endothelial vasomodulatory and antioxidant effects[17,18]. VOO is rich in phenols that are able to prevent endothelial dysfunction by decreasing the expression of cell adhesion molecules[19] and increasing nitric oxide (NO) production[20] by quenching vascular endothelium intracellular free radicals[18]. Olive oil phenolic compounds inhibit platelet-induced aggregation[21] and have been reported to enhance the mRNA transcription of the antioxidant enzyme glutathione peroxidase[20]. It has been shown that inflammatory mediators are actively involved in the cellular damage of cerebral ischemia[22]. The polyphenols from VOO exert an effect of inflammatory processes[23]. VOO consumption was associated with favorable effects on circulating markers; namely, decreased serum TXB2 production[24].

Although in our study VOO increased cholesterol levels, it improved the LDL/HDL ratio – a finding in keeping with those of others[25,26]. It has been identified that high plasma LDL levels are major risk factor for vascular diseases (VD), whereas high levels of HDL are considered a negative risk factor for VD[27]. Oral administration of olive oil induced increased serum triglyceride levels. This may be due to an inhibitory action of oleic acid on VLDL catabolism[28]. VOO analysis of the Zard variety indicated that it is rich in MUFAs (oleic acid: 68.822%). Therefore, high olive oil consumption provides higher amounts of oleic acid, which is responsible for the beneficial effects on health[29,30].

Some criticism has been raised about recommending VOO consumption to protect against cardiovascular diseases because it is presumed to increase the risk of becoming overweight. Our experimental data show that increase in the body weight was not statistically significant.

In conclusion, our data suggest that VOO may be cerebroprotective in a rat model of ischemia-reperfusion. Further work is required to extend these observations. Ultimately, it is hoped that novel cerebroprotective strategies may be developed for those at risk of stroke or in whom cerebral perfusion is electively reduced, perhaps at the time of surgery.

## ACKNOWLEDGMENTS

We thank Professor Hugh Montgomery for critical review on the manuscript. We also thank Banafsheh Mahmoudi, Mahdieh Asheghabadi, and Fatemeh Hemmati for their kind support. This study was supported by Razi Herbal Medicines Research Center, Lorestan University of Medical Sciences and Shahid Beheshti University.
